# Comprehensibility and readability of selected artificial intelligence chatbots in providing uveitis-related information

**DOI:** 10.1097/MD.0000000000045135

**Published:** 2025-10-24

**Authors:** Halil İbrahim Sönmezoğlu, Büşra Güner Sönmezoğlu, Mustafa Hüseyin Temel, Burçin Çakir

**Affiliations:** a Department of Ophthalmology, Hendek State Hospital, Sakarya, Turkey; b Department of Ophthalmology, Serdivan State Hospital, Sakarya, Turkey; c Department of Physical Medicine and Rehabilitation, Sultan 2. Abdulhamid Han Training and Research Hospital, Istanbul, Turkey; d Department of Ophthalmology, Sakarya Training and Research Hospital, Sakarya, Turkey.

**Keywords:** artificial intelligence, ChatGPT-4, readability, understandability, uveitis

## Abstract

This study aims to evaluate and compare the quality and comprehensibility of responses generated by 5 artificial intelligence chatbots – ChatGPT-4, Claude, Mistral, Grok, and Google PaLM – to the most frequently asked questions about uveitis. Google Trends was employed to identify significant phrases associated with uveitis. Each artificial intelligence chatbot was provided with a unique sequence of 25 frequently searched terms as input. The responses were evaluated using 3 distinct tools: The Patient Education Materials Assessment Tool for Printable Materials (PEMAT-P), the Simple Measure of Gobbledygook (SMOG) index, and the Automated Readability Index (ARI). The 3 most frequently searched terms were “uveitis eye,” “anterior uveitis,” and “uveitis symptoms.” Among the chatbots evaluated, GPT-4 demonstrated the lowest ARI and SMOG scores (*P* = .001). Regarding the PEMAT-P, Mistral scored the lowest in understandability, while Grok achieved the highest score for actionability (*P* < .001). All chatbots, except Mistral, exhibited high intelligibility scores. GPT-4 had the lowest SMOG and ARI score among the chatbots evaluated, making it the easiest to read. Chatbot technology holds significant potential to enhance healthcare information dissemination and facilitate better patient understanding. While chatbots can effectively provide information on health topics such as uveitis, further improvement is needed to maximize their efficacy and accessibility.

## 1. Introduction

Uveitis can be a serious cause of vision impairment; thus, it can also reduce the quality of life for the patient.^[1]^ It is estimated that about 5% to 10% of the world population is affected by uveitic vision impairment.^[2]^ In addition, a huge portion of the patient group with uveitis, which accounts for about 35%, sees their vision becoming drastically worse. Some of them are even likely to become permanently blind.^[3,4]^ It is important that patients fully understand the disease process and adhere to treatment at an adequate level. In this context, AI chatbots, which have become increasingly popular in recent times, can also be used to provide information about uveitis and for patient education.

AI chatbots are now widely used for patient education, chronic disease management, mental health support, and self-diagnosis, with evidence of increasing adoption in both developed and developing countries.^[5,6]^

AI chatbots are exceptional robotic programs created for social language-user interactivity. They behave like virtual assistants on forums and web-based applications.^[7]^ These chatbots have many different uses in various sectors, like customer service, healthcare interactions, and symptom identification. In healthcare, AI chatbots help users decide whether to see a doctor.^[8]^

Patient education comprises another main area where AI may be utilized, e.g., giving information about diseases and medication use, offering diagnostic or therapeutic recommendations, patient triage, disclosing the risks and advantages of surgery, and preoperative anxiety management steps along with providing accurate postoperative care and follow-up instructions.^[9]^

Given the level of trust patients have in patient education materials, it is essential that such content be clear and accurate. The information provided by freely accessible AI chatbots is very important, as it enables patients to play an active role in their own healthcare, and therefore must be readable, understandable, and accurate.^[10]^ Therefore, in our study, we examined the quality and readability of the responses provided by 5 different chatbots to keywords related to uveitis obtained from Google Trends (GT).

Google searches exhibit a significant association with current events, particularly emphasizing health and medical inquiries. Public sentiment on healthcare information may also be discerned from GT and has proven particularly beneficial during recent outbreaks and epidemics.^[11]^

Although there are several studies comparing the readability and quality of informative texts on different health conditions and uveitis by AI chatbots, this article aims to compare the comprehensibility, readability, and quality of texts generated by 5 different AI chatbots on frequently asked questions about uveitis obtained with GT and to identify deficiencies, unlike other studies.^[12–14]^

## 2. Methods

The cross-sectional study was conducted on May 1, 2024, in the Ophthalmology Department of Serdivan State Hospital. Our study did not require ethics committee approval since it did not directly collect data through methods such as questionnaires, interviews, focus group studies, did not analyze personal data of people, and did not conduct in vivo or in vitro experiments on subjects.^[15–17]^

GT was used to identify the most frequently searched keywords related to uveitis (https://trends.google.com/). Prior to initiating the searches, all personal browser data were deleted to prevent potential bias. “Uveitis” was entered into GT. Options were selected for all categories worldwide from 2004 to the present. The 25 most frequently searched terms related to the topic were identified. “Uveitis ojo” and “Que es uveitis” were excluded because they were not in English, “Anterior” and “Glaukoma” were excluded because they were not related to Uveitis, and “Uveitis dogs” and “Uveitis in dogs” words were removed because they do not concern the human species. The remaining 19 words were entered separately into each chatbot without being changed. Table 1 shows the 19 most frequent searches about uveitis according to Google trend data from 2004 to 2024.

**Table 1:** The 19 most frequent searches about uveitis according to Google trend data from 2004 to 2024.

Rank	Keyword	Relevance
1	Uveitis eye	100
2	Anterior uveitis	87
3	Uveitis Symptoms	39
4	Uveitis Treatment	38
5	Uveitis eyes	27
6	Uveitis Posterior	25
7	Uveitis in eye	25
8	Uveitis Causes	25
9	What is uveitis	22
10	Iritis	21
11	Uveitis cause	18
12	Uveitis pain	17
13	Arthritis	16
14	Uveitis Ocular	15
15	Acute uveitis	13
16	Uveitis ICD-10	12
17	Uveitis meaning	12
18	Spondylitis	12
19	Ankylosing spondylitis uveitis	10

Search queries were systematically entered into 5 AI chatbots: GPT-4 (https://chat.openai.com/), Claude-3 (https://claude.ai/), Grok (https://grok.x.ai/), Mistral Large (https://mistral.ai/), and Google PaLM 2 (https://ai.google/palm2/). Each query was processed on a separate web page to ensure distinct separation and enhance the analytical process. Individual accounts were created for interacting with each AI chatbot to maintain clear differentiation. Prior to starting the searches, all browser data were thoroughly erased. The chatbot responses were recorded for subsequent evaluation of their quality and clarity.

To evaluate the accuracy and reliability of the healthcare information provided by each chatbot, the Patient Education Materials Assessment Tool for Printable Materials (PEMAT-P) was utilized.^[9]^ Two experienced ophthalmologists, BGS and HIS, jointly discussed and determined the PEMAT-P score. If they cannot reach a joint decision, they consult MHT as a third specialist. The responses of AI chatbots were scored without knowing which bot provided which answers.

The PEMAT-P is applicable to both physical and digital materials. Understandability refers to the ease with which individuals from various backgrounds and levels of health literacy can comprehend, analyze, and articulate the primary message presented in the materials. Actionability assesses how easily consumers can determine the appropriate course of action based on the provided information. The PEMAT-P consists of 24 components, including fifteen items rated on a 2-point scale (0 indicating disagreement and 1 indicating agreement) and 9 items rated on a 3-point scale (0 indicating disagreement, 1 indicating agreement, and 2 indicating the item is not applicable). Higher scores indicate greater understandability or actionability. Materials scoring below 70% are considered to lack understandability or actionability, while scores of 70% or higher indicate the materials are intelligible or actionable.^[18]^ PEMAT-P measures not only the readability of a material, but also how easily patients can understand the information (understandability) and how easily they can apply this information to improve their health (actionability). These 2 dimensions are the cornerstones of the effectiveness of patient education materials. Unlike traditional formulas, PEMAT-P evaluates not only the content itself, but also structural and visual elements. This takes into account how the patient perceives elements such as the layout of the text, font, visual-text relationship, headings, and paragraphs.^[18]^

The simple measure of Gobbledygook (SMOG) index assesses the quantity of words with multiple syllables and the overall number of sentences. The SMOG formula can be calculated using the equation 1.043 multiplied by the square root of the total amount of polysyllabic words, multiplied by 30 divided by the total number of sentences, and then adding 3.1291. The SMOG formula evaluates a text according to the intricacy of its sentences and vocabulary. It specifically examines the quantity of polysyllabic words (words containing 3 or more syllables) and the number of sentences inside a document.^[19]^ It is a readability framework. It measures how many years of education the average person needs to have to understand a text. It is best for texts of 30 sentences or more. Although SMOG is widely used, healthcare is the sector it is mostly used in. Medical use of the formula has been helped along by research. Studies looked at different formulas and their usefulness in healthcare. For example, 1 case study assessing online Parkinson disease information called SMOG the “gold standard.”^[20]^

The automated readability index (ARI) assesses the United States grade level required to read a piece of text. In some ways, it is similar to other formulas. Its difference is rather than counting syllables, it counts characters. The more characters, the harder the word. It also counts sentences. This sets it apart from some other formulas. The ARI calculates a score based on the average number of characters per word and the average number of words per sentence. The ARI is suited to technical writing. The ARI formula can be expressed as 4.71 multiplied by the ratio of the number of characters to the average number of words per phrase, plus 0.5 multiplied by the ratio of the average number of words per sentence to the average number of sentences, minus 21.43.^[19]^ Both SMOG and ARI are part of a group of traditional readability formulas that aim to predict the grade level required to understand a text. Studies show that, although their average scores are generally similar, they can differ by up to 2 grade levels for the same text, especially for technical or complex material. Differences may arise because each formula counts words and sentences differently and processes punctuation marks, abbreviations, and numbers differently.^[21]^

The statistical analysis was conducted using SPSS version 26.0 (IBM, New York, USA). The data’s normality was assessed using the Shapiro–Wilk test. Continuous data were analyzed using the minimum, maximum, mean, median and standard deviation, whereas categorical data were represented by their frequency. The Kruskal–Wallis test was employed to evaluate disparities in means among groups. Bonferroni correction was applied in subgroup analysis. A significance level of .05 was employed, resulting in a confidence interval of 95%.

## 3. Results

**Figure 1.**
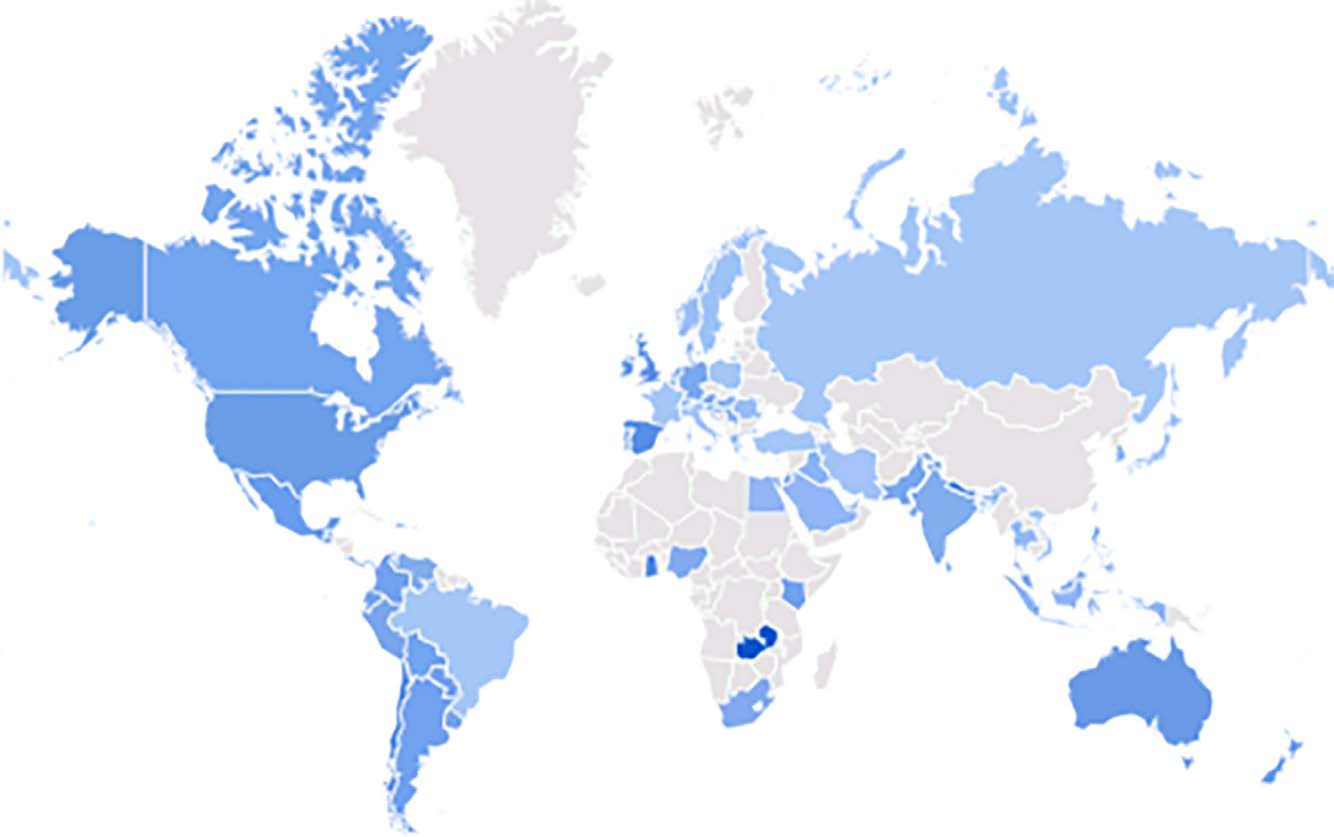
The provided data from Google Trends displays the global search interest in uveitis across different regions from 2004 to 2024. It specifically excludes locations with low search volumes.

The 3 most commonly queried terms were “Uveitis eye,” “Anterior uveitis,” and “Uveitis Symptoms.” (Table 1) Figure 1 shows global search interest in uveitis in different regions from 2004 to 2024, excluding locations with low search volume, using data from GT.

The study’s results indicated a statistically significant difference (*P* < .001) between the SMOG scores of the chatbots. The utilization of the Bonferroni correction statistically significance of these disparities, revealing a statistically significant variation in the SMOG scores between Mistral and Google PaLM, Mistral and ChatGPT-4, Claude-3 and PaLM, and Claude-3 and Chat GPT-4, as well as ChatGPT-4 and Grok. Chat GPT-4 received the lowest score, while Claude-3 received the highest SMOG score (*P* < .001), indicating significant differences in performance.

The study’s findings demonstrated statistically significant differences in the ARI scores across the evaluated chatbots. The application of Bonferroni correction statistical analysis confirmed the statistical significance of these disparities, revealing significant variations in ARI scores between Mistral and Google PaLM, Mistral and ChatGPT-4, Claude-3 and PaLM, Claude-3 and ChatGPT-4, as well as ChatGPT-4 and Grok. ChatGPT-4 attained the lowest ARI score, while Claude-3 achieved the highest score (*P* < .001). So, according to ARI and SMOG scores, Chat GPT-4 had the best readability, while Claude-3 had the worst readability.

The SMOG and ARI scores were found to be strongly correlated. (*P* < 0,001, r:0,946).

The chatbots demonstrated substantial differences in their PEMAT-P scores (*P* < .001). The analysis of PEMAT-P understandability scores, using the Bonferroni correction, revealed that Mistral had significantly lower scores compared to the other chatbots. Furthermore, a notable difference was observed when the chatbots were compared based on their PEMAT-P actionability scores (*P* < .001). Subgroup analysis revealed significant differences, with Claude-3 exhibiting significantly lower scores compared to the other chatbots, and PaLM also showing significantly lower scores relative to the others.

Mean, standard deviation, minimum, maximum, median and interquartile range (IQR) values of PEMAT-A PEMAT-U, SMOG and ARI scores of 5 different chatbots are shown in Table 2.

**Table 2:** Mean, standard deviation, minimum, maximum, median and IQR values of PEMAT-A PEMAT-U, SMOG and ARI scores of 5 different chatbots.

		Mistral	Claude-3	PaLM	GPT-4	Grok	*P*
The Simple Measure of Gobbledygook (SMOG)	Mean ± SD	11.84 ± 1.98	12.35 ± 2.24	9.84 ± 1.78	9.06 ± 1.64	11.20 ± 1.80	**<.001** *
	Min–max	7.15–14.92	8.29–16.56	5.83–12.59	4.06–11.57	8.41–13.88	
	Median	12.48	12.52	9.79	9.45	10.46	
	IQR	(10.2–13.3)	(10.7–14)	(8.7–11.2)	(8.2–10)	(9.7–12.8)	
The Automated Readability Index (ARI)	Mean ± SD	13 ± 2.3	14.22 ± 2.2	11.42 ± 2	10.36 ± 1.83	12.47 ± 1.83	**<.001** †
	Min–max	7–17	11–19	6–15	4–12	10–15	
	Median	14	14	11	11	12	
	IQR	(12–14)	(13–16)	(11–13)	(10–12)	(11–14)	
PEMAT-P Understandability (%)	Mean ± SD	54.31 ± 9.18	75.26 ± 8.3	73.63 ± 6	71.94 ± 9.62	68.74 ± 7.84	**<.001** ‡
	Min–max	40–73	55–82	67–82	58–82	55–82	
	Median	53	80	78	73	67	
	IQR	(46–60)	(70–82)	(67–78)	(64–82)	(64–73)	
PEMAT-P Actionability (%)	Mean ± SD	33.68 ± 14.98	14.73 ± 16.11	3.15 ± 10.02	32.63 ± 11.94	40 ± 14.90	**<.001** §
	Min–max	20–60	0–40	0–40	20–60	20–60	
	Median	40	20	0	40	40	
	IQR	(20–40)	(0–20)	(0–0)	(20–40)	(20–60)	

ARI = automated readability index, GPT = generative pretrained transformer, IQR = interquartile range, PEMAT-p = the patient education materials assessment tool for printable materials, SD = standard deviation, SMOG = simple measure of Gobbledygook index.

* Difference is between mistral and google PaLM, mistral and ChatGPT, claude-3 and PaLM, claude-3 and ChatGPT, ChatGPT and Grok.

† Difference is between mistral and ChatGPT, Claude-3 and PaLM, Claude-3 and ChatGPT, ChatGPT and Grok.

‡ Differences are between mistral and others.

§ Differences between Claude and others, Google PaLM and others.

## 4. Discussion

Our study evaluated the answers provided by 5 different chatbots to questions about uveitis in terms of readability. Notably, none of the chatbots achieved the desired 8th grade reading level.^[22]^ Additionally, the chatbot responses demonstrated low actionability scores on the PEMAT test, while all chatbots except Mistral exhibited high understandability scores.

Given the possibility of leveraging AI to access health information, acquire health-related knowledge, and make treatment decisions, chatbot responses’ readability, quality, and comprehensibility should be of high standards. Health literacy empowers patients to engage in medical decision-making processes actively.^[23]^ Conversely, inadequate readability may impair health literacy, diminishing patients’ capacity to comprehend health information and engage in informed medical decision-making.^[24,25]^ Our study’s analysis of SMOG and ARI metrics revealed that ChatGPT-4 had the best readability, while Claude-3 had the worst. Notably, all the chatbot responses met or exceeded the recommended readability standard. Addressing this challenge may require implementing more advanced natural language processing methods to reduce the complexity of the language. Furthermore, incorporating user feedback mechanisms and algorithms to adjust readability could aid in generating content that is more accessible to the target audience. Several factors contribute to ChatGPT-4’s high readability. These include a comprehensive parameter set, a large number of users and collaborating experts who provide continuous feedback for its training, advanced reasoning and instruction-following capabilities, more up-to-date training data, and insights from the practical applications of previous models integrated into GPT-4’s security research and monitoring system. All of these factors may have contributed to ChatGPT-4 providing more accurate responses.^[16]^

In our study, the most frequently searched words related to uveitis obtained from GT were directly entered into chatbots without any referral questions. Therefore, the quality and readability of the texts may not have been written at the desired levels. With guiding questions, it would also be possible to obtain higher quality and more readable texts. The reason we chose to input such search terms directly was based on the assumption that users might directly ask chatbots these questions.

There are several studies in the literature that have examined the performance of AI systems in ophthalmology. For example, Kianian et al.^[26]^ compared the readability of educational materials on uveitis generated by ChatGPT and Bard, finding that ChatGPT produced more accessible content. Rasmussen et al.^[27]^ found that over half of ChatGPT’s responses to inquiries regarding vernal keratoconjunctivitis were either entirely accurate or contained only negligible and harmless errors. Yilmaz and Dogan comparative analysis revealed that chatbot responses regarding cataracts were more detailed and accurate in content than the information provided on the American Academy of Ophthalmology’s website.^[28]^ Lim et al.’s^[16]^ comparative analysis of chatbot responses related to myopia care specifically highlighted the potential of ChatGPT-4.o to provide accurate and comprehensive answers to myopia-related queries. The cumulative findings from these studies demonstrate that AI chatbots hold significant potential for ophthalmological educational materials despite the current limitations.

There are several studies in the literature about the readability and quality of patient education materials, AI chatbot responses, and online resources in other medical disciplines that have produced findings consistent with the current investigation. For instance, Temel et al.‘s examination of ChatGPT’s responses to frequently asked questions about spinal cord injury revealed that the AI’s outputs lacked sufficient readability and quality.^[29]^ Şahin et al.^[30]^ evaluated the readability and quality of 5 AI-based chatbots focused on erectile dysfunction, finding that none met the required standards. Similarly, Srinivasan et al.^[31]^ compared the readability of patient education materials generated by GPT-3.5, GPT-4, and Bard, as well as online institutional resources, and highlighted the potential of large language models to enhance the readability of bariatric surgery patient education materials. The results of these studies indicate that advanced natural language processing methods to simplify language, user feedback mechanisms to adjust readability, and interdisciplinary collaboration between AI developers, healthcare professionals, and linguists to create more accurate, accessible, and readable content are at the urge.

Chatbots’ Terms of Use and Privacy Policies explicitly state that chatbots are not intended to provide medical advice, diagnosis, or treatment and should not be used as a substitute for professional healthcare. These disclaimers are crucial for understanding the limitations of chatbots’ functionality in the medical domain. Despite this, the natural language fluency of chatbots’ responses can create a false impression of clinical authority, potentially leading users to over-rely on AI-generated information. This disconnect introduces ethical concerns around user safety and informed use. Moreover, the privacy policy cautions users against entering sensitive health information, raising questions about data protection when health-related queries are used in research. From an ethical standpoint, these terms necessitate that researchers emphasize the experimental and informational nature of chatbots, ensuring that neither the study nor its audience misconstrue the tool as a validated clinical support system.^[32,33]^

A low actionability score in chatbots means that they lack clear, practical instructions that users can follow to make informed decisions or take next steps. Studies evaluating AI chatbots’ responses to common cancer-related queries show that actionability scores are consistently low, with median scores ranging from 20% to 40% in validated evaluation tools, suggesting that users may struggle to understand what actions to take based on the chatbot’s recommendations.^[34]^

In our study, although Grok had the highest actionability score, it was still not at the desired level. This limitation is significant because actionable guidance is crucial for effective health communication, especially for patients seeking help with complex medical issues. The use of chatbots with low actionability as a research resource is questionable and should be used as complementary tools rather than primary sources of medical advice. Improving actionability is essential to ensure that chatbots can truly support users in making informed health decisions.

There are several limitations of this study that need to be acknowledged. Firstly, the scope of the analysis was constrained to the initial 25 search terms, which may have restricted the breadth of the study and potentially overlooked other pertinent queries related to uveitis. Secondly, the exclusive focus on English keywords could limit the generalizability of the findings to non-English-speaking populations, potentially reducing the applicability of the results in a global context. Additionally, the study utilized specific AI chatbots available at the time, but as this technology rapidly evolves, newer models may exhibit divergent performance characteristics. Furthermore, the investigation did not account for the varying user experiences and interactions with the chatbots, which could influence perceptions of the quality and readability of the responses. Moreover, as the assessments of the text quality generated by chatbots were performed by 2 ophthalmologists, the outcomes may fluctuate based on the assessors’ expertise, knowledge, and biases.

Future research should consider these factors and explore broader, more inclusive approaches to better understand and enhance the effectiveness of AI chatbots in healthcare education.

## 5. Conclusion

This study revealed statistically significant differences in readability and understandability scores among 5 commonly used chatbots. According to SMOG and ARI, ChatGPT-4 showed the best readability, while Claude-3 showed the poorest readability performance, but none of the chatbots achieved the required reading level. Chatbots other than Mistral demonstrated a high level of understandability. Claude-3 and PaLM received significantly lower actionability scores. These results indicate that AI must be trained by medical literature, utilize neurolinguistic programming techniques, incorporate user feedback mechanisms, and engage in interdisciplinary collaboration to ensure the safe and effective use of AI-based tools in healthcare settings.

## Author contributions

**Conceptualization:** Halil İbrahim Sönmezoğlu.

**Data curation:** Halil İbrahim Sönmezoğlu, Büşra Güner Sönmezoğlu.

**Formal analysis:** Halil İbrahim Sönmezoğlu, Büşra Güner Sönmezoğlu.

**Investigation:** Mustafa Hüseyin Temel.

**Supervision:** Mustafa Hüseyin Temel.

**Writing – original draft:** Halil İbrahim Sönmezoğlu.

**Writing – review & editing:** Mustafa Hüseyin Temel, Burçin Çakir.
